# Genotypic and phenotypic analysis of clinical isolates of *Staphylococcus aureus* revealed production patterns and hemolytic potentials unlinked to gene profiles and source

**DOI:** 10.1186/s12866-016-0630-x

**Published:** 2016-02-01

**Authors:** Andreas Roetzer, Guenter Haller, John Beyerly, Christoph B. Geier, Hermann M. Wolf, Corina S. Gruener, Nina Model, Martha M. Eibl

**Affiliations:** Biomedizinische ForschungsgmbH, Lazarettgasse 19/2, Vienna, A-1090 Austria; Immunology Outpatient Clinic, Schwarzspanierstraße 15, Vienna, A-1090 Austria

**Keywords:** Staphylococcal infection, Exotoxins, Protein quantification, Typing, Hemolysis

## Abstract

**Background:**

Nosocomial infections caused by the bacterial pathogen *Staphylococcus aureus* can lead to serious complications due to the varying presence of secreted toxins. Comparative studies of genomic information and production rates are needed to assess the pathogenic potential of isolated strains. Genotypic and phenotypic profiling of clinical and colonising isolates of *S. aureus* was used to characterise the release of exotoxins. Blood isolates were compared with colonisation strains to determine similarities and differences of single strains and clusters.

**Results:**

Fifty-one fresh isolates obtained from colonised individuals (*n* = 29) and *S. aureus* bacteremia (SAB) patients (*n* = 22) were investigated. The prevalence of genes encoding for three cytolysins (alpha/beta/gamma toxin) and twenty-four superantigens (SEA-SE*l*X) was determined. Isolates exhibited eighteen distinct combinations of superantigens. Sequence analysis identified mutated open reading frames in *hla* in 13.7 % of all strains, in *selw* (92.2 %) and in *selx* (15.7 %). All corrupted genes were associated with specific clonal complexes. Functional assessment of alpha toxin activity by a rabbit erythrocyte lysis assay revealed that supernatants lacking alpha toxin still displayed hemolysis. This was due to the presence of gamma toxin, as proven by inhibition experiments using antisera raised against the respective recombinant proteins. Alpha toxin, SEC, and TSST1 production was quantified by enzyme-linked immunosorbent assays on supernatants of all *hla*, *sec*, and *tst* positive isolates. Blood isolates and colonising strains showed comparable amounts of secreted proteins within a wide range. *Agr* types I to IV were identified, but did not allow a prediction of high or low production rates. In contrast, alpha toxin production rates between distinct clonal complexes clearly differed. *Spa* typing was performed and revealed thirty-two unique *spa* gene patterns and eight small clusters comprising nineteen isolates. Recognised *spa*-typing clusters displayed highly similar production rates.

**Conclusion:**

Production rates of the three most prevalent exotoxins varied within both groups of blood isolates and colonising strains. By comparing genotypes and secretion, we found that identical complex gene patterns did not allow predictions of toxin production and function. However, identification of *spa* typing clusters was suitable to predict similar quantities of released exotoxins.

**Electronic supplementary material:**

The online version of this article (doi:10.1186/s12866-016-0630-x) contains supplementary material, which is available to authorized users.

## Background

The importance of *Staphylococcus aureus* as a human pathogen continued to rise in the first decade of this century [1]. Staphylococcal infections can lead to various, often devastating diseases, such as skin abscesses, osteomyelitis, endocarditis, necrotising pneumonia, sepsis and the toxic shock syndrome (TSS) [2]. Despite an increase of infections due to community-acquired methicillin-resistant *S. aureus* (CA-MRSA) [3], methicillin-sensitive strains (MSSA) still cause the majority of *S. aureus* infections in Western and Northern parts of the European Union [4]. In a long-term study on the epidemiology of TSS in Minnesota (US), MRSA caused only 7 % of all TSS cases [5].

Discussion regarding which virulence factors favor the spread of certain clones in the community is ongoing. Secreted and membrane-associated proteins contribute to virulence of *S. aureus* [6–8]. The majority of virulence factors are encoded in the core genome, which is estimated to include approximately 78 % of the whole genome [9]. However, a prominent group of virulence determinants is sited on vectors such as pathogenic islands (SaPIs) or prophages [10, 11]. Different combinations of these factors might contribute to the broad range of invasion sites and infection outcomes.

The challenging group of exotoxin virulence factors comprises cytolytic toxins and mitogenic superantigen toxins. Members of both have been shown to contribute significantly to the lethal outcome of *S. aureus* infections [12]. The most prominent cytolytic toxin genes encode for pore-forming proteins (alpha toxin and gamma toxin) and are part of the core genome [13]. Alpha toxin is thought to be expressed by all strains, although high and low producers have been described [14]. Similarly, the gamma toxin locus was found in 99 % of all strains [15]. The large group of superantigens (also termed as staphylococcal enterotoxins, SE) is variously spread among clinical isolates [16]. Altogether, twenty-four superantigen genes have been discovered so far. With two exceptions (*selw* and *selx*), superantigens are found on pathogenic islands and prophages. Clinical severity such as sepsis with or without septic shock has been linked to the genomic setup of isolates [17].

Genotypes of clinical strains have been investigated extensively worldwide [18–22]. In contrast, exploration of gene expression and toxin production of clinical isolates is scarce. Transcriptional profiling of superantigens was done in nasal isolates and an outbreak of food poisoning [23, 24]. Protein production of superantigens was analysed in isolates from patients with atopic dermatitis and diabetic foot ulcers [16, 25]. In addition, specific production of SEB, SE*l*K and SEH was quantified in clinical isolates [18, 26, 27]. Protein production of alpha toxin was determined in CA-MRSA strains and in isolates from hemodialysis patients [28–30].

Thorough analysis of the production of virulence factors from obtained clinical isolates could help to recognise potencies of bacteria to lyse host cells or hyperstimulate the immune system. In this study, we performed genomic analyses, as well as quantification and functional analyses of secreted toxins from consecutive isolates collected in large hospitals in Vienna and Linz, Austria. Typing of seven house-keeping genes, the *spa* gene and the *agr* locus was done to link production rates to possible clusters.

## Methods

### Bacterial strains and growth

Bacterial strains were obtained from the general hospital in Vienna and the general hospital in Linz, Austria. As clinical isolates were anonymous and data of the patients were not accessible, the study was exempt from ethical approval, which was testified by the Ethics Committee of the Immunology Outpatient Clinic (www.itk.at/news) after detailed evaluation of the study protocol. Isolates were identified as *S. aureus* by standard laboratory protocols, grown on tryptic soy broth agar plates. Isolates were not subcultured and thereafter stored at −80 °C for further exploration. Bacteremia isolates were designated with the letter B. All strains were identified as MSSA, and genotyped for twenty-four superantigen (−like) genes, four cytolysin genes, the *agr* groups I-IV, and the *spa* gene. All primer pairs are listed in Additional file 1: Table S1. None of the primer pairs reacted with our negative control strain *Staphylococcus epidermidis* ATCC 49461. Genomic DNA templates were purified from overnight cultures according to manufacturer’s protocols using the Wizard Purification Kit (Promega). For cell wall disruption cells were treated with lysostaphin and lysozyme (Sigma). DNA was amplified in a T3 thermocycler (Biometra) by 28 cycles of 95 °C (denaturation) for 30 s, specific annealing temperature for 45 s, and 68 °C (elongation) for 60 s, using the Platinum Taq PCRx DNA polymerase (Invitrogen). The reaction was initiated by 10 min incubation at 95 °C, and terminated by 10 min incubation at 68 °C. Primer sequences and PCR annealing temperatures are shown in Additional file 1: Table S1. Absence of the gene *selv* was verified through analysis of the genes *sei* and *selm*. Distribution statistics were done using the Pearson’s chi-squared test (*χ*^2^).

Isolated genomic DNA of all strains was sequenced using the Illumina MiSeq sequencer, the Nextera XT library kit, and the MiSeq reagent kit as instructed by the manufacturer (Illumina). Upon checking the average size of amplicons using the BioAnalyzer (Agilent), and measuring the concentration using the QuBit system (Life technologies), four genomic libraries were combined for sequencing. Reads were mapped to *S. aureus* gene sequences extracted from GenBank (accession numbers: *sea* NC_003923.1, *seb* NC_002951.2, *sec* KF386012.1, *sed* AF053140.1, *see* M21319.1, *tst* AB678405.1, *seg* NC_009782.1, *seh* NC_002953.3, *sei* NC_009782.1, *selj* AF053140.1, *selk* NC_007793.1, *sell* NC_009782.1, *selm* NC_002745.2, *seln* EF531605.1, *selo* CP002388.1, *selp* NC_002745.2, *selq* NC_017347.1, *selr* AB330135.1, *sels* AB330135.1, *selt* AB330135.1, *selu* AY205307.1, *selu2* EF030428, *selv* EF030427, *selw* CP000046, *selx* CP007447.1, *hla* BX571857.1, *hlg1* S65052.1, *hlg2* S65052.1, *agr* type I AF210055.1, *agr* type II AF001782.1, *agr* type III AF001783, *agr* type IV AF288215.1) using the alignment program CLC (CLCbio, Qiagen). All isolates were sequenced with a minimum coverage of 20-fold. In order to analyse repeat patterns in *spa*, de novo assembly of reads was done using CLC [31]. Blastn of contigs against *spa* (NC002952.2) was performed, and identified gene variants were assigned using the SPATYPEMAPPER software (download at http://www.clondiag.com/fileadmin/Media/Downloads/SPATypeMapper_0_6.zip). All unknown repeat patterns were re-sequenced (Sanger).

Multilocus sequence typing (MLST) was performed as described in [32]. The seven included genes are *arcC*, *aroE*, *glpF*, *gmk*, *pta*, *tpi*, and *ypiL*. Loci information and primer sequences are available on the MLST website, which was also used to analyse all alleles (http://www.mlst.net). New *spa* type and new MLST type were uploaded to the *spa* website (http://www.spaserver.ridom.de) and MLST website. Newly identified *selx* and *hla* mutations have been deposited at GenBank (accession numbers KT943499 and KU236387).

### Protein analysis

For production of superantigens and hemolysins, strains were cultured to stationary phase (16 h) in 25 ml of tryptic soy broth at 37 °C with shaking at 170 revolutions per minute (rpm). Optical densities (OD_600_) of cell cultures were normalised at the beginning of growth (0.02) and compared. In order to receive cell-free supernatants, cultures were centrifuged at 3220 g for five minutes, followed by a sterile filtration of supernatants (PALL Acrodisc 25 mm Syringe Filters with 0.2 μm Posidyne Membrane) as described before [33]. Filtrates were tested for bacterial growth on agar plates. Samples were stored at −20 °C.

Upon addition of sample buffer, supernatants were boiled for three minutes. 20 μl of each sample were resolved on 15 % SDS-polyacrylamide gels. For Western Blotting, proteins were transferred onto 0.2 μm nitrocellulose membranes (GE Healthcare) in a cooled wet blot apparatus (Biorad). Membranes were blocked in a 2 % BSA 1X PBS 0.1 % Tween 20 solution overnight at 4 °C. Antisera used as primary antibodies were employed in a 1:40,000 dilution, secondary antibodies were diluted 1:50,000. Development procedure was performed according to manufacturer’s instructions (GE Healthcare).

### Expression and purification of proteins

Recombinant wild type alpha toxin, gamma toxin, SEC, and TSST1 proteins were produced in our lab. *Escherichia coli* strains (One Shot, Invitrogen) transformed with a pET expression vector (Novagen) carrying hemolysin or superantigen genes, were grown at 28 °C and protein expression was induced by arabinose for 24 h. Pellets from alpha toxin and TSST1 protein expressing bacteria were resuspended in citrate buffer (pH 5), sonicated and centrifuged at 47,000 g. Pellets containing one of the two protein components of gamma toxin, HLG1 or HLG2, were resuspended in phosphate buffer (pH 6.5). The pellet containing the superantigen SEC was resuspended in citrate buffer (pH 5.5). Supernatants were loaded on SP-Sepharose FF columns (GE Healthcare) and proteins were eluted with a NaCl gradient. The peak fraction was dialysed with Tris (pH 8) and applied to Q-Sepharose FF columns (GE Healthcare). The peak fraction was dialysed against 1X PBS and samples were stored at −20 °C.

For the production of antisera, New Zealand White rabbits were purchased from Charles River Laboratories. Animals were kept in standard facilities with free access to water and food (Ssniff), according to the guidelines of the Austrian Ministry for Science and Research. Animal experiments had been approved and controlled by the Veterinary Department of the City of Vienna. Antisera were obtained from rabbits after four rounds of immunisation. Titers were determined through an indirect enzyme-linked immunosorbent assay (ELISA). Flat-bottomed-96-well plates were coated with 0.5 μg per well of wild type recombinant alpha toxin or SEC in carbonate buffer pH 9.6 and further prepared as described in the ELISA section. Plates were incubated with 50 μl per well of samples for 1 h at 37 °C. Horseradish peroxidase-conjugated goat anti-rabbit IgG-HRP antibodies (GE Healthcare) were added in a 1:20,000 dilution. Titers of antisera were determined and expressed as the inverse of the highest dilution (done in twofold dilution steps) for valid detection signals. Antiserum raised against wild type alpha toxin had an ELISA titer of 94,445, wild type SEC antiserum contained a binding titer of 112,382. Antiserum raised against wild type TSST1 had a titer of 46,330 and was described previously by Stich et al. [34]. Polyclonal IgGs were precipitated (37 % ammonium sulfate) from each rabbit antiserum, reconstituted to the initial volume, and stored at −20 °C for further use.

### Hemolysis assay

The assay was adapted from [35, 36]. In detail, a 2 ml sample of blood from rabbits was washed three times with 40 ml of 1X PBS (610 g, 10 min). Washed rabbit erythrocytes were resuspended in 12 ml 1X PBS and verified to have 10^8^ cells per ml using Neubauer counting chambers. The amount of erythrocytes was adjusted to give an optical density of 1.5 at 570 nm (Tecan micro-plate reader) when added to Saponin (Sigma), which was used as a positive control. Staphylococcal supernatants were used in a final dilution of 1:50. Antisera were employed in 1:50, 1:200, and 1:400 dilutions. Samples consisting of supernatants and antisera were incubated in round-bottomed-96-well plates for 1 h at 37 °C. Upon addition of rabbit erythrocytes, samples were incubated at 37 °C for 30 min and afterwards spun down at 610 g for 3 min. 150 μl of supernatant were transferred to a new plate, and the optical density of samples was measured at 570 nm thereafter.

### Enzyme-linked immunosorbent assay

For sandwich enzyme-linked immunosorbent assays (ELISA) monoclonal antibodies were used as capture antibodies, polyclonal antibodies from rabbits were used for detection. In detail, flat-bottomed-96-well plates were coated with 50 μl per well of commercially available monoclonal anti-alpha toxin (MAb6D3, BBI Solutions), anti-TSST1 (MAb5T, BioVeris) or anti-SEC (MAb1C3, BioVeris) antibodies in a dilution of 1:3000 (alpha toxin), 1:1000 (TSST1), or 1:2000 (SEC) in carbonate buffer pH 9.6 and stored at 4 °C. After 16 h of incubation, the plates were washed four times with 1X PBS pH 7.2 containing 0.1 % (v/v) Tween-20. Next, wells were blocked with 200 μl per well using 1X PBS containing 2 % (w/v) BSA and 0.1 % (v/v) Tween-20, and plates were incubated for 1 h at room temperature with gentle agitation. Plates were then stored at −20 °C.

Standards, samples (supernatants) and controls were diluted in blocking buffer and plates were incubated with 100 μl per well at room temperature for 90 min with 150 revolutions per minute. The plates were then washed four times and 100 μl per well of polyclonal IgG preparations from rabbit antisera in a dilution of 1:15,000 (alpha toxin) or 1:7500 (TSST1, SEC) in blocking buffer were added. After incubation for 90 min at room temperature, plates were again washed three times. Goat anti-rabbit IgG-HRP antibodies (GE Healthcare) were added in a 1:5000 dilution (100 μl per well) for 1 h at room temperature and continuous movement (150 revolutions per minute). An o-phenylendiamine pastille (Sigma) was suspended in substrate buffer. H_2_O_2_ was added to catalyse the reaction, which was kept in the dark at room temperature. To stop this colorimetric reaction, 100 μl of a 1 % (v/v) sulfuric acid solution were added per well. The plates were scanned for absorbance at 492 nm wavelength (Tecan micro-plate reader).

## Results

### Analysis of toxins genes

In this study, the distribution and expression of genes encoding superantigens and hemolysins was explored in 51 fresh clinical and colonisation isolates. 22 strains were derived from patients with bacteremia, 29 isolates were collected from the nasal cavity of healthy individuals. The prevalence of four cytolysin genes and 24 superantigen genes was determined in all strains by singleplex-PCR amplification and whole genome sequencing (Additional file 2: Table S2).

Since both hemolysins, alpha toxin and gamma toxin belong to the core genome, we detected these genes (*hla*, *hlg1*, *hlg2*) in all strains. Despite the fact, that *hla* was found in all strains by PCR, sequence analysis revealed, that ten isolates (19.6 %) had a known nonsense mutation leading to a shortened alpha toxin protein [37]. The majority of isolates (80.4 %) showed a corrupted beta toxin gene *hlb*, based on the presence of the phage φSa3. In contrast, gamma toxin genes *hlg1* and *hlg2* were present and in frame in all isolates.

The genomic distribution of all known superantigen and superantigen-like genes was further analysed (Additional file 2: Table S2). The latest discovered superantigen-like genes *selw* and *selx* were found in 51 isolates (100 %) and 39 isolates (76.5 %), respectively. Importantly, sequence analysis revealed the lack of a start codon in *selw* in the vast majority of isolates and only four strains displayed a functional open reading frame of *selw* (7.8 %). In addition, a yet unknown one base pair deletion has been detected in *selx* right after the start at position twelve in eight isolates (15.7 %). The resulting frameshift led to a stop codon in *selx* at codon position eleven. This means that 20 out of 51 isolates (39.2 %) did not express SE*l*X due to the corruption or absence of the gene. Further, two strains had a shorter version of *selu*, which has already been described as *selu2* [38].

**Fig. 1:**
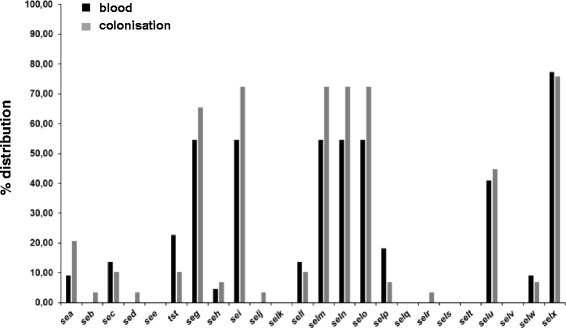
Prevalence of superantigen and superantigen-like genes in blood isolates and colonisation strains. Percentage of distribution of genes identified by PCR was determined, *selw* was included when sequencing revealed an ATG start codon

Genotypic analysis did not reveal a correlation between gene profiles and the source of isolates in this study. Two superantigen encoding genes (*tst*, *selp*) were more often found in blood isolates (Fig. 1). However, the difference between blood isolates and colonising isolates was not statistically significant (Table 1). Pseudogene versions of *selw* were excluded from this analysis. Further, comparison of isolates having no superantigen or superantigen-like genes at all (with the exception of *selw* and *selx*), resulted in an even distribution (n_b_ = 6, n_c_ = 5; *P* = 0.44) as well.

**Table 1:** Prevalence of superantigen and superantigen-like open reading frames in blood and colonising isolates

	All (*n* = 51)^a^	Blood (*n* = 22)^a^	Colonising (*n* = 29)^a^	*P*-value^b^
*sea*	8 (15.7)	2 (9.1)	6 (20.7)	0.26
*seb*	1 (1.9)	0 (0)	1 (3.4)	nd
*sec*	6 (11.8)	3 (13.6)	3 (10.3)	0.72
*sed*	1 (1.9)	0 (0)	1 (3.4)	nd
*see*	-	-	-	
*tst*	8 (15.7)	5 (22.7)	3 (10.3)	0.23
*seg*	31 (60.8)	12 (54.5)	19 (65.5)	0.43
*seh*	3 (5.9)	1 (4.5)	2 (6.9)	0.72
*sei*	33 (64.7)	12 (54.5)	21 (72.4)	0.19
*selj*	1 (1.9)	0 (0)	1 (3.4)	nd
*selk*	-	-	-	
*sell*	6 (11.8)	3 (13.6)	3 (10.3)	0.72
*selm*	33 (64.7)	12 (54.5)	21 (72.4)	0.19
*seln*	33 (64.7)	12 (54.5)	21 (72.4)	0.19
*selo*	33 (64.7)	12 (54.5)	21 (72.4)	0.19
*selp*	6 (11.8)	4 (18.2)	2 (6.9)	0.22
*selq*	-	-	-	
*selr*	1 (1.9)	0 (0)	1 (3.4)	nd
*sels*	-	-	-	
*selt*	-	-	-	
*selu* ^*c*^	21 (41.2)	8 (36.4)	13 (44.8)	0.54
*selv*	-	-	-	
*selw* ^*d*^	4 (7.8)	2 (9.1)	2 (6.9)	0.77
*selx*	39 (76.4)	17 (77.3)	22 (75.8)	0.91

^a^No. of PCR-amplified and sequenced genes, percentages are in parentheses

^b^Pearson’s chi-squared test (*χ* ^2^) performed for distribution of in-frame genes (*n* > 1)

^c^ *selu* and *selu2* combined as *selu*

^d^ *selw* with identified ATG as start codon

To assess clonality of strains, *spa* typing was performed with all 51 isolates (Table 2). We identified 39 types, among which 30 types were unique (58.8 %). 21 strains were identified to belong to one of nine clusters. The largest cluster comprised four strains (*spa* type t084), which belonged to the group of isolates without prominent superantigens. One cluster contained three isolates (t091), showing an identical superantigen and cytolysin gene pattern. Six clusters were found to contain only two isolates each (displaying the repeat patterns t056, t015, t021, t342, t246, t433). These pairs had identical superantigen and cytolysin gene patterns, however, in one cluster (t056), *hlb* was found to be corrupted in only one isolate.

**Table 2:** Results for testing the presence of superantigens, cytolysins and the measurement of protein amounts in hospital-derived isolates

Isolates			Typing		Superantigens	Cytolysins	α toxin	SEC	TSST
		IS^b^	*spa*	*agr*			[μg/ml]		
**no superantigens** ^**a**^	Rv53944	c	t377	IV	*selw* ^*e*^ *, selx*	*hla, hlb, hlg1, hlg2*	25.4	-	-
	B7715	b	t024	I	*selw* ^*e*^ *, selx*	*hla, hlb, hlg1, hlg2*	11.45	-	-
	B3276	b	t056	I	*selw* ^*e*^ *, selx*	*hla, hlb, hlg1, hlg2*	6.12	-	-
	B3478	b	t056	I	*selw* ^*e*^ *, selx*	*hla, hlg1, hlg2*	5.4	-	-
	Rv54213	c	t648	I	*selw* ^*e*^ *, selx*	*hla, hlb, hlg1, hlg2*	4.83	-	-
	Rv52743	c	t084	II	*selw* ^*e*^ *, selx*	*hla, hlg1, hlg2*	1.37	-	-
	Rv54192	c	t084	II	*selw* ^*e*^ *, selx*	*hla, hlg1, hlg2*	0.43	-	-
	771 N-10	c	t084	II	*selw* ^*e*^ *, selx*	*hla, hlg1, hlg2*	0.15	-	-
	B7761	b	t084	II	*selw* ^*e*^ *, selx*	*hla, hlg1, hlg2*	0.10	-	-
	B5990	b	t008	I	*selw* ^*e*^ *, selx*	*hla, hlg1, hlg2*	0.05	-	-
	B50188	b	t2453	II	*selw* ^*e*^ *, selx*	*hla, hlg1, hlg2*	0.05	-	-
***sec***	B958	b	t790	I	*sec, sell, egc, selw* ^*e*^ *, selx*	*hla, hlg1, hlg2*	36.13	19.71	-
	Rv52832	c	t583	I	*sec, sell, egc, selw* ^*e*^ *, selx* ^*f*^	*hla, hlg1, hlg2*	7.44	5.77	-
	Rv51398	c	t230	I	*sec, sell, egc* ^*d*^ *, selw* ^*e*^ *, selx* ^*f*^	*hla, hlg1, hlg2*	6.05	11.12	-
	876 N-10	c	t333	I	*sec, sell, egc* ^*d*^ *, selw* ^*e*^ *, selx* ^*f*^	*hla, hlg1, hlg2*	2.72	1.95	-
	B1721	b	t015	I	*sec, sell, egc* ^*d*^ *, selw* ^*e*^ *, selx* ^*f*^	*hla, hlg1, hlg2*	2.75	6.93	-
	B3427	b	t015	I	*sec, sell, egc* ^*d*^ *, selw* ^*e*^ *, selx* ^*f*^	*hla, hlg1, hlg2*	1.75	5.79	-
***tst***	B2284	b	t067	II	*tst, selp, egc* ^*d*^ *, selw, selx*	*hla, hlg1, hlg2*	0.99	-	2.83
	B1848	b	t021	III	*tst, egc, selw* ^*e*^ *, selx*	*hla* ^*g*^ *, hlb, hlg1, hlg2*	≤0.01	-	3.71
	B1793	b	t021	III	*tst, egc, selw* ^*e*^ *, selx*	*hla* ^*g*^ *, hlb, hlg1, hlg2*	≤0.01	-	3.24
	840 N-10	c	t342	III	*tst, sea, egc, selw* ^*e*^ *, selx*	*hla* ^*g*^ *, hlg1, hlg2*	≤0.01	-	0.5
	B34571	b	t342	III	*tst, sea, egc, selw* ^*e*^ *, selx*	*hla* ^*g*^ *, hlg1, hlg2*	≤0.01	-	0.85
	B11019	b	t15407^c^	III	*tst, seh, egc, selw* ^*e*^	*hla* ^*g*^ *, hlg1, hlg2*	≤0.01	-	2.66
	Rv52959	c	t012	III	*tst, sea, egc, selw* ^*e*^ *, selx*	*hla* ^*g*^ *, hlg1, hlg2*	≤0.01	-	0.06
	Rv54054	c	t3805	III	*tst, seh, egc, selw* ^*e*^	*hla* ^*g*^ *, hlb, hlg1, hlg2*	≤0.01	-	0.01
***egc***	Rv53955	c	t246	IV	*egc, selw* ^*e*^ *, selx*	*hla, hlg1, hlg2*	6.58	-	-
	Rv54216	c	t246	IV	*egc, selw* ^*e*^ *, selx*	*hla, hlg1, hlg2*	2.88	-	-
	784 N-10	c	t065	I	*egc* ^*d*^ *, selw* ^*e*^ *, selx* ^*f*^	*hla, hlg1, hlg2*	8.26	-	-
	Rv51334	c	t13078	I	*egc* ^*d*^ *, selw* ^*e*^ *, selx*	*hla, hlg1, hlg2*	11.64	-	-
	Rv51410	c	t617	III	*egc, selw* ^*e*^	*hla* ^*g*^ *, hlg1, hlg2*	≤0.01	-	-
	Rv51412	c	t11980	III	*egc, selw* ^*e*^	*hla* ^*g*^ *, hlg1, hlg2*	≤0.01	-	-
	Rv52825I	c	t005	I	*egc* ^*d*^ *, selw* ^*e*^ *, selx*	*hla* ^*h*^ *, hlg1, hlg2*	0.02	-	-
	Rv52825II	c	t350	I	*egc* ^*d*^ *, selw* ^*e*^ *, selx* ^*f*^	*hla, hlg1, hlg2*	3.99	-	-
	B7709	b	t3232	I	*egc, selw* ^*e*^ *, selx*	*hla, hlg1, hlg2*	1.85	-	-
	B8186	b	t553	I	*egc, selw* ^*e*^ *, selx* ^*f*^	*hla, hlg1, hlg2*	4.55	-	-
	Rv54035	c	t284	IV	*egc, selw* ^*e*^ *, selx*	*hla, hlb, hlg1, hlg2*	2.87	-	-
	B16586	b	t4401	III	*egc, selw* ^*e*^	*hla* ^*g*^ *, hlg1, hlg2*	≤0.01	-	-
	B1455	b	t071	II	*egc* ^*d*^ *, selw, selx*	*hla, hlg1, hlg2*	1.01	-	-
	803 N-10	c	t1441	IV	*egc, selw* ^*e*^ *, selx*	*hla, hlb, hlg1, hlg2*	28.23	-	-
**diverse**	Rv54209	c	t548	II	*egc* ^*d*^ *, selp, selw, selx*	*hla, hlb, hlg1, hlg2*	0.29	-	-
	869 N-10	c	t002	II	*egc* ^*d*^ *, selp, selw, selx*	*hla, hlg1, hlg2*	2.01	-	-
	B69108	b	t091	I	*selp, selw* ^*e*^ *, selx*	*hla, hlg1, hlg2*	1.73	-	-
	B24743	b	t091	I	*selp, selw* ^*e*^ *, selx*	*hla, hlg1, hlg2*	0.69	-	-
	B3597	b	t091	I	*selp, selw* ^*e*^ *, selx*	*hla, hlg1, hlg2*	1.52	-	-
	Rv54009	c	t433	III	*sea, egc, selw* ^*e*^ *, selx*	*hla, hlg1, hlg2*	1.58	-	-
	Rv54010	c	t433	III	*sea, egc, selw* ^*e*^ *, selx*	*hla, hlg1, hlg2*	1.45	-	-
	Rv52745	c	t3802	I	*sea, selw* ^*e*^ *, selx*	*hla, hlg1, hlg2*	2.44	-	-
	B3155	b	t3308	I	*sea, selw* ^*e*^ *, selx*	*hla, hlg1, hlg2*	2.16	-	-
	767 N-10	c	t008	I	*sea, selw* ^*e*^ *, selx*	*hla, hlg1, hlg2*	3.80	-	-
	Rv51379	c	t6873	II	*seb, seh, egc, selw* ^*e*^ *, selx*	*hla, hlg1, hlg2*	0.31	-	-
	638 N-10	c	t878	III	*sed, sej, selr, selw* ^*e*^ *, selx*	*hla, hlg1, hlg2*	7.34	-	-

^a^except *selw* and *selx*

^b^Isolation Site including (b) blood isolates and (c) colonising strains

^c^new, unique repeat pattern 04-54-31-12-16-34-16-12-25

^d^no *selu*

^e^due to the absence of the atg start codon designated as pseudogene

^f^frameshift nonsense mutation in *selx* (I11)

^g^nonsense mutation in *hla* (Q113)

^h^frameshift nonsense mutation in *hla* (M317) plus a different C-terminus E315K E316K

Assessment of *agr* types revealed the presence of all four types in this study (Table 2). Type I was most prevalent (*n* = 23, 45.1 %), whereas type II and III were found in ten (19.6 %) and thirteen (25.5 %) isolates, respectively. Type IV was present in five strains (9.8 %). There was no significant correlation between *agr* types and isolation site. Strains within each *spa* type cluster displayed identical *agr* types. Among the groups of *tst*-positive and *sec*-positive strains, only one *agr* type was predominant: type I was found in all *sec*-positive isolates, type III was present in seven out of eight *tst*-positive strains. All other groups defined through superantigen patterns displayed an even distribution of *agr* types.

Further, MLST was performed to investigate the dispersion of clonal complexes among blood samples and nasal isolates (Table 3). In addition, identified *spa* types and *agr* loci were linked to present clonal complexes, and distribution of superantigen and superantigen-like genes was assessed. In total, fourteen clonal complexes were found, with CC30, which comprised twelve isolates, being the most prevalent one. Interestingly, identified valid start codons of *selw* were restricted to CC5, whereas the nonsense mutation of *selx* was linked to CC45. MLST of the strain 767 N-10, which displayed *spa* type t008, revealed a new single locus variant (SLV) of sequence type 8 (ST8), with a different allele of *aroE*. In far the most cases, identical *spa* types belong to the same clonal complex. Here, we identified a peculiar deviation, t008 was found in ST8 (B5990) and the newly discovered variant ST3275 (767 N-10). Both STs also differed in their superantigen gene patterns.

**Table 3:** Characterisation of clonal complexes and comparison of alpha toxin production rates

Clonal complex	ST (n)	*spa* (n)	*agr* (n)	Superantigens^b^	Production (m/sd)^d^ α toxin
CC5	ST5 (3)	t071, t548, t002	II (3)	*selp* (2)*, egc* (3)*, selw* (3), *selx* (3)	1.1 (+/−0.7)
	ST1457 (1)	t067	II	*tst, selp, egc, selw, selx*	
CC6	ST6 (1)	t3802	I	*sea, selx*	
ST7	ST7 (3)	t091 (3)	I (3)	*selp* (3), *selx* (3)	1.32 (+/−0.55)
CC8	ST8 (4)	t3308, t024, t008, t648	I (5)	*sea* (1), *selx* (4)	8.78 (+/−10.23)
	ST3275 (1)^a^	t008		*sea* (1), *selx* (1)	
	ST630 (1)	t377	IV	*selx*	
CC10	ST10 (1)	t6873	II	*seb, seh, egc, selx*	
CC15	ST15 (2)	t2453, t084	II (5)	*selx* (2)	0.42 (+/−0.55)
	ST582 (3)	t084 (3)		*selx* (3)	
CC22	ST22 (3)	t790, t13078, t005	I (3)	*sec* (1)*, sell* (1)*, egc* (3), *selx* (3)	15.93 (+/−18.43)
CC25	ST25 (1)	t3232	I	*egc, selx*	
CC30	ST30 (10)	t433 (2), t4401, t11980, t617, t012, t021 (2), t342 (2)	III (10)	*tst* (5)*, sea* (5)*, egc* (10), *selx* (10)	≤0.01
	ST34 (2)	t3805, t15407	III (2)	*tst* (2)*, seh* (2)*, egc* (2), *selx* (2)	
CC45	ST45 (8)	t015 (2), t333, t230, t583, t065, t350, t553	I (8)	*sec* (5)*, sell* (5)*, egc* (8), *selx* (8)^c^	4.69 (+/−2.36)
CC50	ST50 (2)	t246 (2)	IV (2)	*egc* (2), *selx* (2)	
CC51	ST121 (2)	t284, t1441	IV (2)	*egc* (2), *selx* (2)	
CC101	ST101 (2)	t056 (2)	I (2)	*selx* (2)	
CC779	ST779 (1)	t878	III	*sed, sej, selr, selx*	

^a^Strain 767 N-10 displayed a different allele of *aroE*, being a new single locus variant of ST8

^b^All superantigen genes were included with the exception of pseudogenes

^c^Superantigen-like gene *selx* including frameshift nonsense mutation (I11)

^d^Mean (m) and standard deviation (sd) of production rates were determined of CC groups comprising one or more STs (number of strains n ≥ 3)

### Protein expression of bacterial toxins

Concentrations of alpha toxin, SEC, and TSST1, were determined by ELISA, using commercially available monoclonal antibodies (see Table 2). First, we assessed amounts of alpha toxin in all supernatants grown to stationary phase and compared concentrations of this toxin between distinct groups. Due to the fact that *hla* was present in all strains, isolates were grouped a priori based on the distribution of superantigens (Table 2, first column). Among the panel of strains bearing no prominent superantigen gene, all supernatants displayed measurable amounts of alpha toxin. Interestingly, we found an up to 500-fold difference in alpha toxin concentration between supernatants. Two strains produced significantly higher amounts: Rv53944 (25.4 μg/ml) and B7715 (11.45 μg/ml). 0.05 μg/ml was the lowest amount of alpha toxin found in the supernatants of two blood isolates (B5990 and B50188).

Within the group of *sec*-positive strains, the divergence between secreted alpha toxin amounts was less pronounced. However, the supernatant with the highest amount of alpha toxin produced, belonged to this group: B958 with 36.1 μg/ml. Among the group of *tst*-positive strains, we found seven strains bearing the nonsense mutation in *hla* (7/8, 87.5 %). In these strains, concentration of alpha toxin was below the detection limit of 0.01 μg/ml. The only isolate with a functional alpha toxin among these strains (B2284, sole *agr* type II) showed an amount of 0.99 μg/ml. Characterisation of strains from the two remaining groups of egc strains and variant strains revealed similar, wide-ranging alpha toxin concentrations.

Thereafter, we determined the amount of secreted SEC and TSST1 (Table 2). Analysis of the respective protein production revealed a broad range of production rates. All six strains bearing *sec* were able to produce significant amounts of SEC. However, there was a ten-fold difference between the highest (19.71 μg/ml) and lowest (1.95 μg/ml) amount measured. We also found varying TSST1 concentrations among *tst*-positive strains. Six out of eight samples showed one-digit μg values. Two strains stepped out of the line: the nasal isolates Rv52959 and Rv54054, which were both identified to carry the intact *tst* gene, produced only minimal amounts of TSST1 (0.06 μg/ml and 0.01 μg/ml, respectively).

Comparison of production rates and *agr* types did not unravel the broad range of concentrations of superantigens or alpha toxin. Of note, among the small group of *agr* type IV strains, all produced alpha toxin in μg ranges. All isolates bearing the *hla* mutation were found to have *agr* type III. In contrast, comparison of alpha toxin production rates revealed noteworthy differences between clonal complexes (see Table 3, last column). We found clonal complexes with rates around 1 μg per ml (CC5, ST7), clonal complexes displaying much higher average amounts (CC8, CC22, CC45), and clonal complexes showing much lower amounts (CC15, and CC30). However, sample sizes between clonal complexes differed considerably. Strikingly, *spa* typing of isolates revealed a high similarity of production rates among identical *spa* types and exotoxin gene patterns (Table 4). These similarities within *spa* type clusters were found throughout all clonal complexes. The two members of the *sec*-positive cluster produced almost identical quantities of SEC, the two pairs of *tst*-positive isolates showed highly similar amounts of TSST1 in their supernatants. In addition, within the eight *spa* typing clusters, quantification of alpha toxin revealed related amounts.

**Table 4:** Comparison of protein amounts in *spa* typing clusters with identical superantigen and alpha toxin gene patterns of hospital-derived isolates

		Typing			α toxin	SEC	TSST1
	IS	*spa*	*agr*	CC	[μg/ml]		
B3276	b	t056	I	101	6.12	-	-
B3478	b	t056	I		5.4	-	-
Rv52743	c	t084	II	15	1.37	-	-
Rv54192	c	t084	II		0.43	-	-
771 N-10	c	t084	II		0.15	-	-
B7761	b	t084	II		0.10	-	-
B1721	b	t015	I	45	2.75	6.93	-
B3427	b	t015	I		1.75	5.79	-
B1848	b	t021	III	30	≤0.01	-	3.71
B1793	b	t021	III		≤0.01	-	3.24
840 N-10	c	t342	III	30	≤0.01	-	0.5
B34571	b	t342	III		≤0.01	-	0.85
Rv53955	c	t246	IV	50	6.58	-	-
Rv54216	c	t246	IV		2.88	-	-
B69108	b	t091	I	ST7	1.73	-	-
B24743	b	t091	I		0.69	-	-
B3597	b	t091	I		1.52	-	-
Rv54009	c	t433	III	30	1.58	-	-
Rv54010	c	t433	III		1.45	-	-

### Functional assessment of hemolysis

**Fig. 2:**
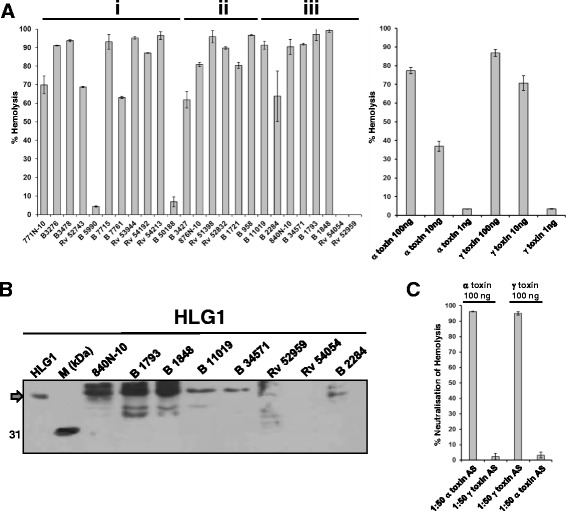
Hemolysis of erythrocytes through supernatants from *S. aureus* depends on alpha toxin and gamma toxin. **a** To measure percentage of hemolysis, cells were grown to stationary phase, and sterile-filtered supernatants were diluted 1:50 in 1X PBS. Absolute hemolysis was defined as the optical density of freed heme upon the addition of the natural glycoside saponin (from *Quillaja saponaria*) and was set at 100%. Each run of hemolysis was accompanied by parallel measurement of saponin as positive control and 1X PBS as negative control. Percentages were calculated from mean values out of three independent experiments. Groups i) to iii) are defined in the text. Recombinant wild type alpha toxin and recombinant wild type gamma toxin were diluted in 1X PBS to receive 100 ng, 10 ng, and 1 ng per well. For neutralisation, 100 ng of toxin was incubated with or without 50-fold diluted antisera (AS) in parallel for 1 h at 37 °C. **b** Western blot analysis of HLG1 protein levels in the supernatants of *tst*-positive isolates. 20 μl were taken from each supernatant (sn) and loaded undiluted. 0.1 μg/μl of recombinant wild type HLG1 was used as positive control (lane 1, grey arrow). Broad range standard (Biorad) was added to identify correct bands (lane 2). Upon blotting, membrane was cut to avoid extensive background. **c** Neutralisation of hemolysis of erythrocytes through antisera was assessed. To verify specificity, antiserum against alpha toxin or gamma toxin was used as control for the opposite hemolysin (100 ng per well). Antisera were applied in a final 50-fold dilution and incubated for 1 h at 37 °C. Hemolysis and percentage calculation (here for neutralisation of hemolysis) was done as described in (**a**)

Alpha toxin quantification confirmed the expression of *hla* in the majority of strains. Assessment of functional activity of alpha toxin in all supernatants to lyse freshly prepared rabbit erythrocytes revealed hemolytic activities in corresponding strains (data not shown). However, surprisingly, all *tst*-positive isolates having the *hla* nonsense mutation displayed hemolysis. Therefore, based on varying alpha toxin production rates, we chose to compare hemolysis of strains within three major, homogeneous groups of isolates defined through distinct superantigen and cytolytic gene patterns. Strains identified as *tst*-positive showed no alpha toxin production (with one exception), *sec*-positive isolates displayed high amounts of alpha toxin in their supernatants, whereas strains without prominent superantigens showed a very broad variation of production (see Table 2). Strains were divided into three sets i) having no superantigens (*n* = 11), ii) having *sec* (*n* = 6), and iii) having *tst* (*n* = 8) (Fig. 2a). Within the first group, all supernatants displayed hemolysis. The two blood isolates that showed the lowest alpha toxin production (B5990, B50188), also showed the weakest hemolysis. All supernatants of the *sec*-positive strains showed hemolytic activity as well. Surprisingly, in the group of *tst*-positive isolates, five supernatants displayed hemolysis despite the absence of full-length alpha toxin. Only two *tst*-positive colonising samples (Rv52959, Rv54054) showed no hemolysis.

We explored the reason for the hemolytic activity of all *tst*-positive strains lacking functional *hla* in more detail. Beside alpha toxin, pore-forming gamma toxin lyses blood cells as well. To assess the putative presence of gamma toxin in the *tst*-positive samples, we performed western blotting with all *tst*-positive strains for HLG1, one essential monomer of gamma toxin (Fig. 2b). Despite a high background level due to the Fc binding capacity of certain *S. aureus* supernatants, five isolates which had displayed hemolysis before, were positive for HLG1. The two colonising isolates (Rv52959 and Rv54054), which did not perform any hemolysis at all, did not show HLG1 protein. Thereafter, we determined activity of recombinant wild type alpha toxin and recombinant wild type gamma toxin (Fig. 2a right panel). We were able to detect hemolysis at toxin concentrations as low as 10 ng per well.

Then we selectively blocked hemolysis of supernatants in this assay by applying alpha toxin and gamma toxin antisera from rabbits immunised with recombinant proteins (Table 5). Both antisera blocked only hemolysis of the specific hemolysin, thereby reaching neutralisation levels of 95 % (Fig. 2c). In the first group (upper panel), supernatants had to be neutralised by antisera against both, alpha toxin and gamma toxin, while other supernatants could be neutralised with antiserum against alpha toxin alone. Interestingly, the two isolates of one *spa* type cluster, which also differed in the presence of the intact *hlb* gene, differed in the neutralisation patterns of hemolysis: the supernatant of B3478 had to be neutralised with both antisera, whereas the supernatant of B3276 was neutralised with antiserum against alpha toxin alone. In the *sec*-positive group, it was necessary to apply both antisera, indicating that all supernatants contained both, alpha toxin and gamma toxin. In the group consisting of *tst*-positive strains, supernatants of five strains lacking full length alpha toxin were neutralised by gamma toxin antiserum alone, while the remaining supernatant (B2284) was neutralised by alpha toxin alone. This underlined the extraordinary potency of both, alpha and gamma toxin, to harm cells of the host.

**Table 5:** Analysis of hemolytic performance of hemolysins upon neutralisation in hospital-derived isolates

Isolates		Cytolysins	>90 % Inhibition of hemolysis		
			α toxin AS^c^	γ toxin AS^c^	α/γ toxin AS^c^
**no superantigens** ^**a**^	Rv53944	*hla, hlb, hlg1, hlg2*			1:50/1:200
	B7715	*hla, hlb, hlg1, hlg2*			1:100/1:200
	B3478	*hla, hlg1, hlg2*			1:400/1:400
	Rv54213	*hla, hlb, hlg1, hlg2*			1:400/1:200
	Rv54192	*hla, hlg1, hlg2*			1:400/1:400
	B3276	*hla, hlb, hlg1, hlg2*	1:400		
	Rv52743	*hla, hlg1, hlg2*	1:400		
	B50188	*hla, hlg1, hlg2*	1:400		
	771 N-10	*hla, hlg1, hlg2*	1:400		
	B5990	*hla, hlg1, hlg2*	1:400		
	B7761	*hla, hlg1, hlg2*	1:400		
***sec***	B958	*hla, hlg1, hlg2*			1:50/ 1:400
	Rv52832	*hla, hlg1, hlg2*			1:400/1:200
	Rv51398	*hla, hlg1, hlg2*			1:400/1:200
	B1721	*hla, hlg1, hlg2*			1:400/1:400
	876 N-10	*hla, hlg1, hlg2*			1:400/1:400
	B3427	*hla, hlg1, hlg2*			1:400/1:400
***tst***	B2284	*hla, hlg1, hlg2*	1:400		
	B11019	*hla* ^*b*^ *, hlg1, hlg2*		1:200	
	840 N-10	*hla* ^*b*^ *, hlg1, hlg2*		1:400	
	B34571	*hla* ^*b*^ *, hlg1, hlg2*		1:200	
	B1848	*hla* ^*b*^ *, hlb, hlg1, hlg2*		1:200	
	B1793	*hla* ^*b*^ *, hlb, hlg1, hlg2*		1:200	
	Rv52959	*hla* ^*b*^ *, hlg1, hlg2*	-	-	-
	Rv54054	*hla* ^*b*^ *, hlb, hlg1, hlg2*	-	-	-

^a^except *selw* and *selx*

^b^nonsense mutation in *hla* (Q113)

^c^ *AS* antiserum

## Discussion

Comprehensive studies of genotypes and phenotypes of clinical *S. aureus* isolates are scarce. To contrast differences between SAB isolates and colonising strains, we performed MLST, *spa* and *agr* typing, we determined the presence of four hemolysin genes and 24 superantigen genes in 22 blood and 29 colonising isolates, and thereafter compared production of alpha toxin, and the two prominent superantigens, TSST1 and SEC. Allocation of genes and production rates of exotoxins was equally distributed between blood and colonising samples. However, corrupted genes and production patterns were linked to certain clonal complexes. *Spa* typing was found to be a suitable predictor for highly similar toxin production rates.

The prevalence of genes encoding superantigens or superantigen-like proteins was both abundant and diverse among blood and nasal isolates. In 2004, a German multicenter study including 429 isolates from 32 hospitals revealed 73 % of all samples studied to be SE positive [19]. Among 51 isolates, we found 78 % to carry common superantigen genes. In accordance with the majority of previous reports, we did not see any correlation between superantigen distribution and isolation site [18, 19]. We assume that the presence or absence of superantigens and cytolysins does not decide about the success of colonisation. In this line, subsequent bacteremia is likely to be more dependent on host factors than the arsenal of exotoxins [39]. Still, we cannot exclude that these virulence factors can facilitate invasion at specific entry sites. The severity of disease upon invasion such as pneumonia or endocarditis has been shown to be effected by the presence of prominent superantigens in rabbit models [40, 41].

All tested isolates contained at least two staphylococcal enterotoxin-like genes, since both, *selw* and *selx* were found in all strains, even those lacking any other superantigen. S*elw* was identified in all strains, whereas *selx* was found in 76.5 % of isolates. Importantly, we found that more than 90 % of isolates had no corresponding start codon at the correct position in *selw,* questioning its relevance as a putative virulence factor. However, we could not exclude the use of alternative codons as transcription start site. The exact role of SE*l*W remains to be determined [42]. Interestingly, *selx* was found to be mutated in seven isolates. Thus functional SE*l*X was lacking in 39.2 % of all isolated strains. Wilson and colleagues discovered *selx* in 95 % of 114 tested isolates [43]. To our knowledge, our study was the first to include both, *selw* and *selx*, in genotypic analysis of clinical and colonisation isolates.

*Agr* typing revealed the presence of all four types. Homogeneous distribution was found in *sec*-positive isolates (type I), and in *tst*-positive isolates (type III), as previously reported [44]. All *agr* types were equally distributed in the remaining isolates. *Spa* typing uncovered 30 unique strains whereas the minority of strains were linked to small clusters. Epidemiological data from contact tracing information were not available. Genotypic information of isolates clustered by MLST and *spa* typing was identical, with one exception. The disruption of *hlb* was reversed in the second isolate of the paired cluster with the *spa* repeat pattern t056. Phage excision resulting in reintroduction of beta toxin production has already been shown [45].

Differences in hemolytic activity regarding TSS isolates were already reported in 1982 [46, 47]. The involvement of alpha toxin was shown a few years later [48, 49], the specific nonsense stop mutation also detected in our strains was described in 1990 [37]. In our study, highly variable expression rates of alpha toxin could be detected in both blood and colonising isolates. In fact, alpha toxin production by individual blood strains varied up to 700-fold. This might be due to variations in the concentration of the regulator RNAIII, or the lack of host cell stimulators [50]. In our study, analysis of *agr* typing revealed that all strains having the *hla* mutation belonged to *agr* type III. Low producers were also found in *agr* types I and II. Remarkably, 20 % of all strains produced less that 1 μg per ml of alpha toxin. Analysis of the *agrC* sequence of the two isolates producing less than 0.1 μg per ml (B5990, B50188) did not reveal the already described *agrC*_G55R_ single nucleotide polymorphism [51]. Small *spa* clusters were found among alpha toxin high and low producers, and isolates having the *hla* mutation. Spaulding and colleagues hypothesised that the reduction of alpha toxin production in USA200 strains facilitates colonisation of mucosal surfaces [2]. In a recent publication, Sharma-Kuinkel et al. describe that the absence of functional alpha toxin was associated with a negative clinical outcome, indicating the clinical relevance of other virulence factors [30]. In their study, 14.5 % of 200 tested strains showed no alpha toxin production. Here, we found that 19.6 % of all isolates lacked alpha toxin production.

Overall, high-level alpha toxin secretion does not seem to be mandatory for the colonisation of the human host by *S. aureus*, since strains lacking *hla* expression are continuously isolated from patients. Long-term persistence might be easier to achieve through the decrease of the overall burden caused by secreted exotoxins. Compromised body defence mechanisms could be a major reason for those strains to enter the bloodstream and organs. It was already reported before that CC30 isolates, which are often associated with severe infection, accumulate the *hla* nonsense mutation [51]. In our study, CC30 *tst*-positive clones displayed complete absence of functional Hla. Amounts of alpha toxin varied between the identified clonal complexes with CC22 displaying the highest yield of Hla. CC22 was found to be associated with osteoarticular infections but was negatively associated with persistent bacteremia [39, 52]. Further quantifications of exotoxins are needed to confirm their relevance for persistence, invasion and severity of disease. It remains to be clarified whether in vitro toxin production rates reflect the situation in the host. Comparison of alpha toxin production in vitro and in an infective endocarditis model in rabbit revealed the production of significant amounts in both settings [53].

Alpha toxin is still thought to be one of the most important candidates of *S. aureus* for vaccine development, clinical studies test monoclonal antibodies against alpha toxin as primary targets for treatment. However, the role of gamma toxin should not be underestimated, our results suggest that gamma toxin is of importance for toxicity in the absence of *hla* expression. Alpha toxin and gamma toxin act at least partly on the same target cells [13, 54]. It was shown that both promote virulence in a murine model [55]. Both, alpha toxin and gamma toxin were found to activate caspase-1 in the presence of bacterial lipoprotein, ultimately leading to necrotic cell death [56]. Synergy of alpha toxin and gamma toxin could facilitate survival in the host. In our study, the necessity to neutralise both alpha toxin and gamma toxin, to block hemolysis in 44 % of all tested isolates, demonstrated this functional redundancy. The specific role of gamma toxin for the lysis of human erythrocytes was just recently shown by Spaan et al. [57].

Availability of both monoclonal antibodies and polyclonal antisera allowed us to further characterise TSST1 and SEC. Blood isolates and colonising isolates contained high and low producers. We did not see a concordance between production rates of superantigens and production rates of alpha toxin. Varshney et al. determined the expression rate of *seb* in *S. aureus* isolates [18]. They also found that SEB production varied greatly among individual strains grown under identical conditions. When we compared expression rates of SEC within the genetically homogeneous *sec*-positive group, we found an up to 10-fold difference. Among the eight *tst*-positive isolates, we found four distinct superantigen patterns. Accordingly, results of these strains varied even more. Nevertheless, weak expression of superantigens may still be a potential threat for host cells [58]. Beside the lack of genotypic differences between colonising strains and blood isolates, production rates of superantigens did not distinguish between them either. For example, the two *sec*/*sell* strains with the highest production rates of SEC displayed identical complex gene patterns (with the exception of *selu2*), but were isolated from both, the nasal cavity and blood. We suggest that fatality of superantigens comes into play when *S. aureus* manages to enter the bloodstream and organs [59].

Importantly, in both groups, SAB and colonisation, the small clusters based on *spa* typing displayed similar production rates. Even though not surprising, this correlation of *spa* types and specific production rates has not been shown before. In contrast, identical complex gene patterns, including both superantigen encoding genes located on various insertion sites and cytolysin encoding genes belonging to the core genome, gave no information about expectable production rates of the tested exotoxins. Despite being a single-locus typing technique, analysis of the polymorphic repeats within the *spa* gene gave a sufficient resolution to identify clusters of high or low producers of specific toxins. Thus, identical virulence gene patterns could not predict production rates, but *spa* typing was suitable for correct predictions, regardless of the isolation site.

## Conclusions

Quantitative and functional characterisation of toxins supports the understanding of clinical relevance. In this study, we compared distribution and secretion of *S. aureus* exotoxins of fresh isolates. Genotypic analysis revealed that all superantigen genes were in frame with the exception of the two new superantigen genes located in the core genome, *selw* and *selx*, showing unexpectedly high corruption rates, which were linked to specific clonal complexes. Identical genotypic characteristics did not allow a quantitative prediction for secretion. Essentially, we discovered that strains within each of the *spa* typing clusters produced highly similar amounts of the respective toxins in supernatants. Despite a broad range, we found that clonal complexes displayed distinct alpha toxin production rates. A high prevalence of both the *hla* nonsense mutation and *tst* was found in CC30. Phenotypic analysis showed that both, alpha toxin and gamma toxin participated, albeit to different degrees, in the hemolytic activity detected in culture supernatants. In addition to genotypic characterisation, we consider further quantification of varying toxin productions in bacterial populations to be clinically important for the development of efficient treatments.

## Availability of supporting data

Supporting data provided as additional files include all primer sequences and annealing temperatures for superantigen and hemolysin gene singleton PCR. Further, an overview table with all according identified genes is provided. Newly identified mutations are available at the GenBank database (accession numbers KT943499 and KU236387). New *spa* types and MLST types were uploaded to the *spa* website (http://www.spaserver.ridom.de) and MLST website (http://www.mlst.net) and are freely accessible.

## Additional files

Additional file 1: Table S1. Primer Sequences and Annealing Temperatures. (PDF 357 kb) Additional file 2: Table S2. Genomic Setup of Superantigens and Cytolysins of 51 clinical isolates. (PDF 460 kb)

## Abbreviations

CA-MRSA: Community-acquired methicillin-resistant *Staphylococcus aureus*
HLA: Hemolysin alpha
SAB: *Staphylococcus aureus* bacteremia
MSSA: Methicillin-sensitive *Staphylococcus aureus*
MLST: Multi locus sequence typing
SE: Staphylococcal enterotoxin
SE*l*: Staphylococcal enterotoxin-like
SLV: Single locus variant
TSS: Toxic shock syndrome
TSST1: Toxic shock syndrome toxin 1
