# Supplementary material for: Genotypic and phenotypic analysis of clinical isolates of Staphylococcus aureus revealed production patterns and hemolytic potentials unlinked to gene profiles and source
Source: BMC Microbiol. 2016 Feb 1;16:13. doi: 10.1186/s12866-016-0630-x (PMC4736648; doi:10.1186/s12866-016-0630-x)
Supplement: Additional file 1: Table S1. — Primer Sequences and Annealing Temperatures. (PDF 357 kb) [file 12866_2016_630_MOESM1_ESM.pdf]

Additional file 1: Table S1: Primer Sequences and Annealing Temperatures

Targets	Forward primer	Reverse primer	Annealing	Source
sea	CAGCATACTATATTGTTTAAAG	TCTTAATAGTGTATTTGAATTCT	56°C	this study
seb	TGCACAAATCGAGTAAATTC	TCACTTTTTCTTTGTCGTAA	52°C	this study
sec	agatttagcaaagaagtacaaagatg	aaggtggacttctatcttcacactt	45°C	[1]
sed	TCTGAATTAAGTAGTACCGC	ATTCGTAATTGTTTTTCGGG	53°C	this study
see	cttggttcaaaagatgctactaa	ctattaattcagagaacctcc	60°C	[2]
tst	TAAAGGATTTGCTAGACTGG	GGTGGTTTTTCAGTATTGTAT	52°C	this study
seg	GTTGAAGGAAGAGGAGTTAT	TCAACAACTTTATTATCTCCG	52°C	this study
seh	TAGCTAATGCATATGGTCAA	AGATTTTAAAGTTTTATTGTCTTCA	53°C	this study
sei	CTATTGCAAATCAACTCGAA	AAAAACTTACAGGCAGTCC	52°C	this study
sej	ACGAAAAGGGTATCTCTGAA	ACAGAACCAAAGGTAGACTT	54°C	this study
selk	AGGAATTGATAATCTCAGGA	CCAAATGGAATTTCTCAGAC	52°C	this study
sell	AAAATTCACCAGAATCACAC	TTAAGAAGCTTTCTGGAAGA	52°C	this study
selm	GATAATTCGACAGTAACAGC	CGACAGTTTTGTTGTCATTA	52°C	this study
seln	TACTGATATAACGTGGCAAT	AGATGAGCTAACTGTTCTATT	52°C	this study
selo	TTGTGTAAGAAGTCAAGTGT	GATAGTCTGATGAATCTATTGTT	53°C	this study
selp	GACCTTGGTTCAAAAGACACC	TGTCTTGACTGAAGGTCTAGC	52°C	this study
selq	AAAAGCTTCAAGGAGTTAGT	ATCCAAATGAAAATTCTCTGC	53°C	this study
selr	agcggtaatagcagaaaatg	tcttgtaccgtaaccgtttt	55°C	[3]
sels	TGAATTAGATTCAACCGCAC	CGTCTATGTGTAAATTTGAAGAG	54°C	this study
selt	CGAATCAATACATTAGACGA	TTGTGTAATCAAGTGTAAAGT	51°C	this study
selu	aatggctctaaaattgatgg	atttgatttccatcatgctc	55°C	[3]
selw	ATTTTTACAAATTCAGCGAGT	TAACACCACCATAACTACAT	52°C	this study
selx	TTACGATAGATACAGCAAGG	TGTCATTAACACTTTTCACAA	52°C	this study
hla	ACTACAGATATTGGAAGCAA	TTTCTTCTTTTTCCCAATCG	52°C	this study
hlb	AAGCCGAATCTAAGAAAGAT	TTTCAGTCACAACTTCATTG	52°C	this study
hlg1	GGTAAAATAACACCAGTCAG	TTGTGATTTTCCCAATCAAT	52°C	this study
hlg2	CATCAAAAGAACACAAGACA	TTACTTAGGTGTGATGCTTT	52°C	this study
spa	agacgatccttcggtgagc	gcttttgcaatgtcatttactg	50°C	[4]

References

1. Couch JL, Soltis MT, Betley MJ: Cloning and nucleotide sequence of the type E staphylococcal enterotoxin gene. J Bacteriol 1988, 170:2954–2960.

2. Sergeev N, Volokhov D, Chizhikov V, Rasooly A: Simultaneous Analysis of Multiple Staphylococcal Enterotoxin Genes by an Oligonucleotide Microarray Assay. J Clin Microbiol 2004, 42:2134–2143.

3. Varshney AK, Mediavilla JR, Robiou N, Guh A, Wang X, Gialanella P, Levi MH, Kreiswirth BN, Fries BC: Diverse enterotoxin gene profiles among clonal complexes of *Staphylococcus aureus* isolates from the Bronx, New York. Appl Environ Microbiol 2009, 75:6839–6849.

4. Frénay HM, Bunschoten AE, Schouls LM, van Leeuwen WJ, Vandenbroucke-Grauls CM, Verhoef J, Mooi FR: Molecular typing of methicillin-resistant *Staphylococcus aureus* on the basis of protein A gene polymorphism. Eur J Clin Microbiol Infect Dis Off Publ Eur Soc Clin Microbiol 1996, 15:60–64.
