# Supplementary material for: Genotypic and phenotypic analysis of clinical isolates of Staphylococcus aureus revealed production patterns and hemolytic potentials unlinked to gene profiles and source
Source: BMC Microbiol. 2016 Feb 1;16:13. doi: 10.1186/s12866-016-0630-x (PMC4736648; doi:10.1186/s12866-016-0630-x)
Supplement: Additional file 2: Table S2. — Genomic Setup of Superantigens and Cytolysins of 51 clinical isolates. (PDF 460 kb) [file 12866_2016_630_MOESM2_ESM.pdf]

**Additional file 2: Table S2. Genomic Setup of Superantigens and Cytolysins of 51 clinical isolates.**

| Isolates | Superantigens | | | | | | | | | | | | | | | | | | | | | | | | Cytolysins | | | |
|---|---|---|---|---|---|---|---|---|---|---|---|---|---|---|---|---|---|---|---|---|---|---|---|---|---|---|---|---|
| | a | b | c | d | e | ts | g | h | i | j | k | l | m | n | o | p | q | r | s | t | u | v | w | x | α | β | γ1 | γ2 |
| 771N-10 | | | | | | | | | | | | | | | | | | | | | | | # | ocher | green | ° | green | green |
| B3276 | | | | | | | | | | | | | | | | | | | | | | | # | ocher | green | green | green | green |
| B3478 | | | | | | | | | | | | | | | | | | | | | | | # | ocher | green | ° | green | green |
| Rv52743 | | | | | | | | | | | | | | | | | | | | | | | # | ocher | green | ° | green | green |
| B 5990 | | | | | | | | | | | | | | | | | | | | | | | # | ocher | green | ° | green | green |
| B7715 | | | | | | | | | | | | | | | | | | | | | | | # | ocher | green | green | green | green |
| B7761 | | | | | | | | | | | | | | | | | | | | | | | # | ocher | green | ° | green | green |
| Rv53944 | | | | | | | | | | | | | | | | | | | | | | | # | ocher | green | green | green | green |
| Rv54192 | | | | | | | | | | | | | | | | | | | | | | | # | ocher | green | ° | green | green |
| Rv54213 | | | | | | | | | | | | | | | | | | | | | | | # | ocher | green | green | green | green |
| B50188 | | | | | | | | | | | | | | | | | | | | | | | # | ocher | green | ° | green | green |
| B3427 | | | red | | | | orange | | orange | | | red | orange | orange | orange | | | | | | | | # | ocher " | green | ° | green | green |
| 876N-10 | | | red | | | | orange | | orange | | | red | orange | orange | orange | | | | | | | | # | ocher " | green | ° | green | green |
| Rv51398 | | | red | | | | orange | | orange | | | red | orange | orange | orange | | | | | | | | # | ocher " | green | ° | green | green |
| Rv52832 | | | red | | | | orange | | orange | | | red | orange | orange | orange | | | | | | orange ^ | | # | ocher " | green | ° | green | green |
| B1721 | | | red | | | | orange | | orange | | | red | orange | orange | orange | | | | | | | | # | ocher " | green | ° | green | green |
| B958 | | | red | | | | orange | | orange | | | red | orange | orange | orange | | | | | | orange ^ | | # | ocher | green | ° | green | green |
| Rv54054 | | | | | | red | orange | red | orange | | | | orange | orange | orange | | | | | | orange | | # | | green * | green | green | green |
| B11019 | | | | | | red | orange | red | orange | | | | orange | orange | orange | | | | | | orange | | # | | green * | ° | green | green |
| B2284 | | | | | | red | orange | | orange | | | | orange | orange | orange | red | | | | | | | ocher | ocher | green | ° | green | green |
| 840N-10 | red | | | | | red | orange | | orange | | | | orange | orange | orange | | | | | | orange | | # | | green * | ° | green | green |
| B34571 | red | | | | | red | orange | | orange | | | | orange | orange | orange | | | | | | orange | | # | | green * | ° | green | green |
| Rv52959 | red | | | | | red | orange | | orange | | | | orange | orange | orange | | | | | | orange | | # | | green * | ° | green | green |
| B1793 | | | | | | red | orange | | orange | | | | orange | orange | orange | | | | | | orange | | # | | green * | green | green | green |
| B1848 | | | | | | red | orange | | orange | | | | orange | orange | orange | | | | | | orange | | # | | green * | green | green | green |
| 638N-10 | | | | red | | | | | | red | | | | | | | | red | | | | | # | ocher | green | ° | green | green |
| Rv51379 | | red | | | | | orange | red | orange | | | | orange | orange | orange | | | | | | orange | | # | ocher | green | ° | green | green |
| 767N-10 | red | | | | | | | | | | | | | | | | | | | | | | # | ocher | green | ° | green | green |
| B3155 | red | | | | | | | | | | | | | | | | | | | | | | # | ocher | green | ° | green | green |
| Rv52745 | red | | | | | | | | | | | | | | | | | | | | | | # | ocher | green | ° | green | green |
| Rv54009 | red | | | | | | orange | | orange | | | | orange | orange | orange | | | | | | orange | | # | | green | ° | green | green |
| Rv54010 | red | | | | | | orange | | orange | | | | orange | orange | orange | | | | | | orange | | # | | green | ° | green | green |
| B3597 | | | | | | | | | | | | | | | | red | | | | | | | # | ocher | green | ° | green | green |
| B24743 | | | | | | | | | | | | | | | | red | | | | | | | # | ocher | green | ° | green | green |
| B69108 | | | | | | | | | | | | | | | | red | | | | | | | # | ocher | green | ° | green | green |
| 869N-10 | | | | | | | orange | | orange | | | | orange | orange | orange | red | | | | | | | ocher | ocher | green | ° | green | green |
| Rv54209 | | | | | | | orange | | orange | | | | orange | orange | orange | red | | | | | | | ocher | ocher | green | green | green | green |
| Rv53955 | | | | | | | | | orange | | | | orange | orange | orange | | | | | | orange | | # | ocher | green | ° | green | green |
| Rv54216 | | | | | | | | | orange | | | | orange | orange | orange | | | | | | orange | | # | ocher | green | ° | green | green |
| 784N-10 | | | | | | | orange | | orange | | | | orange | orange | orange | | | | | | | | # | ocher " | green | ° | green | green |
| Rv51334 | | | | | | | orange | | orange | | | | orange | orange | orange | | | | | | | | # | ocher | green | ° | green | green |
| Rv51410 | | | | | | | orange | | orange | | | | orange | orange | orange | | | | | | orange | | # | | green * | ° | green | green |
| Rv51412 | | | | | | | orange | | orange | | | | orange | orange | orange | | | | | | orange | | # | | green * | ° | green | green |
| Rv52825I | | | | | | | orange | | orange | | | | orange | orange | orange | | | | | | | | # | ocher | green [1] | ° | green | green |
| Rv52825II | | | | | | | orange | | orange | | | | orange | orange | orange | | | | | | | | # | ocher " | green | ° | green | green |
| B7709 | | | | | | | orange | | orange | | | | orange | orange | orange | | | | | | orange | | # | ocher | green | ° | green | green |
| B8186 | | | | | | | orange | | orange | | | | orange | orange | orange | | | | | | orange | | # | ocher " | green | ° | green | green |
| Rv54035 | | | | | | | orange | | orange | | | | orange | orange | orange | | | | | | orange | | # | ocher | green | green | green | green |
| B16586 | | | | | | | orange | | orange | | | | orange | orange | orange | | | | | | orange | | # | | green * | ° | green | green |
| B1455 | | | | | | | orange | | orange | | | | orange | orange | orange | | | | | | | | ocher | ocher | green | ° | green | green |
| 803N-10 | | | | | | | orange | | orange | | | | orange | orange | orange | | | | | | orange | | # | ocher | green | green | green | green |

superantigens = red; egc superantigens = orange; *selw* and *selx* = ocher; cytolytic toxins = green

^ = *selu2*

# = pseudogene

" = 1bp deletion

* = nonsense mutation

[1] = altered C-terminus

° = phage disruption
